# Nystagmus Does Not Limit Reading Ability in Albinism

**DOI:** 10.1371/journal.pone.0158815

**Published:** 2016-07-08

**Authors:** Muriel Dysli, Mathias Abegg

**Affiliations:** Department of Ophthalmology, Inselspital, Bern University Hospital, University of Bern, Bern, Switzerland; The University of Melbourne, AUSTRALIA

## Abstract

**Purpose:**

Subjects with albinism usually suffer from nystagmus and reduced visual acuity, which may impair reading performance. The contribution of nystagmus to decreased reading ability is not known. Low vision and nystagmus may have an additive effect. We aimed to address this question by motion compensation of the nystagmus in affected subjects and by simulating nystagmus in healthy controls.

**Methods:**

Reading speed and eye movements were assessed in 9 subjects with nystagmus associated with albinism and in 12 healthy controls. We compared the reading ability with steady word presentation and with words presented on a gaze contingent display where words move in parallel to the nystagmus and thus correct for the nystagmus. As the control, healthy subjects were asked to read words and texts in steady reading conditions as well as text passages that moved in a pattern similar to nystagmus.

**Results:**

Correcting nystagmus with a gaze contingent display neither improved nor reduced the reading speed for single words. Subjects with nystagmus and healthy participants achieved comparable reading speed when reading steady texts. However, movement of text in healthy controls caused a significantly reduced reading speed and more regressive saccades.

**Conclusions:**

Our results argue against nystagmus as the rate limiting factor for reading speed when words were presented in high enough magnification and support the notion that other sensory visual impairments associated with albinism (for example reduced visual acuity) might be the primary causes for reading impairment.

## Introduction

Albinism is a common clinical entity with an estimated prevalence of 1:17000 [1]. It is a heterogeneous [2], autosomal recessive or X-linked inherited disease caused by deficient melanin synthesis due to mutations resulting in different types of either oculocutaneous albinism (OCA1-4; OCA1A being the most severe type) [3], or ocular albinism (OA, X-chromosomal) [4]. Albinism of the eyes is clinically characterized by deficient or absent pigment in the fundus and iris. The latter may manifest with iris transillumination. Additional clinical signs are foveal hypoplasia, fundus hypopigmentation, refractive errors [3,5], reduced contrast sensitivity [6–9], colour vision impairment, photophobia [1], amblyopia, and sometimes the use of a non-foveal retinal locus for fixation [10]. The optic chiasm usually contains fewer uncrossed optic nerve fibers [11], which might lead to impaired binocularity and/or strabismus [12]. A characteristic finding in subjects with albinism is nystagmus, which in 25–30% [13] up to 40% [14] can be of the periodic alternating type, i.e. it changes direction every few minutes [15]. Subjects with albinism typically have reduced visual acuity, ranging from 20/60 to 20/400 [1] due to light interference [8], refractive errors, nystagmus, and/or foveal hypoplasia. The refractive errors can simply be detected and corrected. Additionally, optical coherence tomography (OCT) allows to grade the level of foveal hypoplasia in subjects with albinism [16]. This allows a prognosis for the visual acuity. However, the contribution of nystagmus to decreased visual acuity is more difficult to determine. A major complaint of subjects with albinism is difficulty with reading [17–19]. Merrill et al. measured the reading acuity, i.e. the smallest size of print that the subjects can resolve, the maximum reading speed, and the critical print size, i.e., the smallest print that the subjects can read with maximum speed [17–19]. They found that reading acuity is reduced in children with albinism. Woo & Bedell (2006) reported ~20% slower reading speeds in subjects with infantile nystagmus, compared to normal subjects [20]. Barot et al. reported that reading speed was similar in subjects with idiopathic infantile nystagmus and subjects with nystagmus associated with albinism [21]. Reading in both groups was approximately 15% slower than in normal control subjects. From this, they concluded that maximum reading speed could be near normal in subjects with infantile nystagmus when optimal font size is provided, even in individuals with poor visual acuity or intense nystagmus. However, they measured significantly more errors in subjects with albinism than in subjects with idiopathic infantile nystagmus or controls [22]. So while it seems clear that reduced visual acuity itself limits reading performance, it is less clear whether the nystagmus associated movements of gaze on a text may additionally impair reading speed.

In this study we investigated the contribution of nystagmus eye movements to reading. For this purpose we measured reading performance of subjects with albinism and nystagmus while reading texts and single words that were corrected for the nystagmus by the use of a gaze contingent display. Moreover, we measured reading speed of healthy subjects who read moving words and texts with a movement pattern recorded from a subject with nystagmus. These results were compared to reading of non-moving (steady) texts in healthy controls and affected subjects.

## Materials and Methods

### Subjects

Nine subjects with clinically diagnosed ocular or oculocutaneous albinism and nystagmus (4 female; median age 17 years, range 12 to 46) and 12 healthy subjects without nystagmus (7 female; median age 23.5 years, range 14 to 26) participated. All participants were native Swiss German speakers and all subjects with albinism were tested with best refractive correction (5 with glasses, 2 with contact lenses, 2 without correction). One of the healthy control subjects had a high hyperopia (+7 diopters) corrected with contact lenses; the rest of the controls were emmetropic. Best corrected visual acuity (BCVA) was measured at 6 meters and Snellen visual acuity was subsequently converted to logMAR (logarithm of Minimum Angle of Resolution) for further analysis. The main characteristics of all subjects with albinism are presented in Table 1. None of the subjects with albinism showed manifest strabismus; subject 5 had strabismus surgery years ago. The ‘null-position’ (i.e. the viewing direction with the least marked nystagmus) was not determined and subjects were not allowed to adopt a head posture during eye movement recording (see also below). The study was conducted with approval of the local ethics committee Bern (‘Kantonale Ethikkommission Bern’ (KEK)), Switzerland, and all subjects gave informed written consent in accordance with the Declaration of Helsinki. For subjects under 18, written consent was obtained in parallel from one of the parents.

**Table 1:** Characteristics of subjects with nystagmus (BCVA = best corrected visual acuity, * = with minimal foveating saccade, MLN 4 = manifest latent nystagmus type 4 (i.e. persistent in binocular viewing) [23], INS = infantile nystagmus syndrome).

subject no	age	sex	correction	BCVA decimal	BCVA logMAR	dominant waveform
1	12	f	glasses	0.63	0.2	jerk, MLN 4
2	15	m	-	0.4	0.4	pendular *
3	17	f	glasses	0.4	0.4	jerk, MLN 4
4	17	f	glasses	0.63	0.2	periodic alternating
5	17	m	glasses	0.2	0.7	pendular
6	23	m	glasses	0.16	0.8	jerk-pendular *
7	27	m	-	0.12	0.9	-
8	30	f	contact lens	0.4	0.4	pendular
9	46	m	contact lens	0.32	0.5	jerk, INS

### Experimental setup

Experiments were conducted using a video-based eye-tracking system (EyeLink1000, SR Research). The subjects’ head was stabilized with a chin- and a forehead-rest. Stimuli (isolated words and continuous text paragraphs) were presented on a 19” CRT screen (Viewsonic Graphics Series G220fb) with a spatial resolution of 1024x768 pixels and a refresh rate of 100 Hertz (Hz). The desktop-mount camera was positioned at 50 cm from the subjects’ forehead, leading to a subject—screen—distance of 60 cm. The screen spanned a visual angle of 41.2 degrees (°) horizontally and 31.5° vertically. Horizontal and vertical eye movements of both eyes were recorded with an infrared camera at a sample rate of 1000 Hz for each eye. Prior to the experiment, the eyetracker was calibrated binocularly with a nine-point-horizontal-vertical calibration grid. Calibration was accepted if the default calibration procedure indicated accuracy better than 1°. Before each of the two blocks, accuracy of calibration was tested for the central position and recalibration was done, if necessary. In one subject with albinism (no 7) calibration was not possible due to marked nystagmus. In this subject the calibration from a healthy subject (MD) was used. Preliminary experiments in healthy subjects showed that this calibration resulted in an error of about one degree. For ‘gaze contingent’ display, the right eye was always taken as the template eye. Thus the stimulus on the CRT screen was positioned at the actual gaze position of the right eye. This method was used despite the fact that some groups found the nystagmus eye movements to be highly similar but not identical in the two eyes of subjects with infantile nystagmus [24]. Ocular dominance was not assessed. A further spatial error may originate from the temporal delay from gaze recording until the stimulus position on the screen is updated: If we assume a peak velocity of 300° per second during a saccade, the maximal spatial error resulting from the temporal delay of our experimental hardware, which is determined by the screen refresh rate, would be 3°. Given the phenomenon of suppression during a saccade we considered this spatial error insignificant. We assumed the spatial error associated with gaze contingent display to be an order of magnitude lower during slow and more relevant eye movements, i.e. below one degree, and thus within the range of the intended accuracy.

The subject’s voice was recorded with a microphone that was attached to the forehead-rest. After calibration the chin-rest was removed to allow mobility of the lower jaw while speaking, however, subjects were instructed to retain exactly the same position and the forehead-rest remained for head stability. Subjects with nystagmus were thus forced to read the words in primary position and were not allowed to adopt their null-position, i.e. the position with the least marked nystagmus.

#### Experimental protocol for word reading

**Fig 1:**
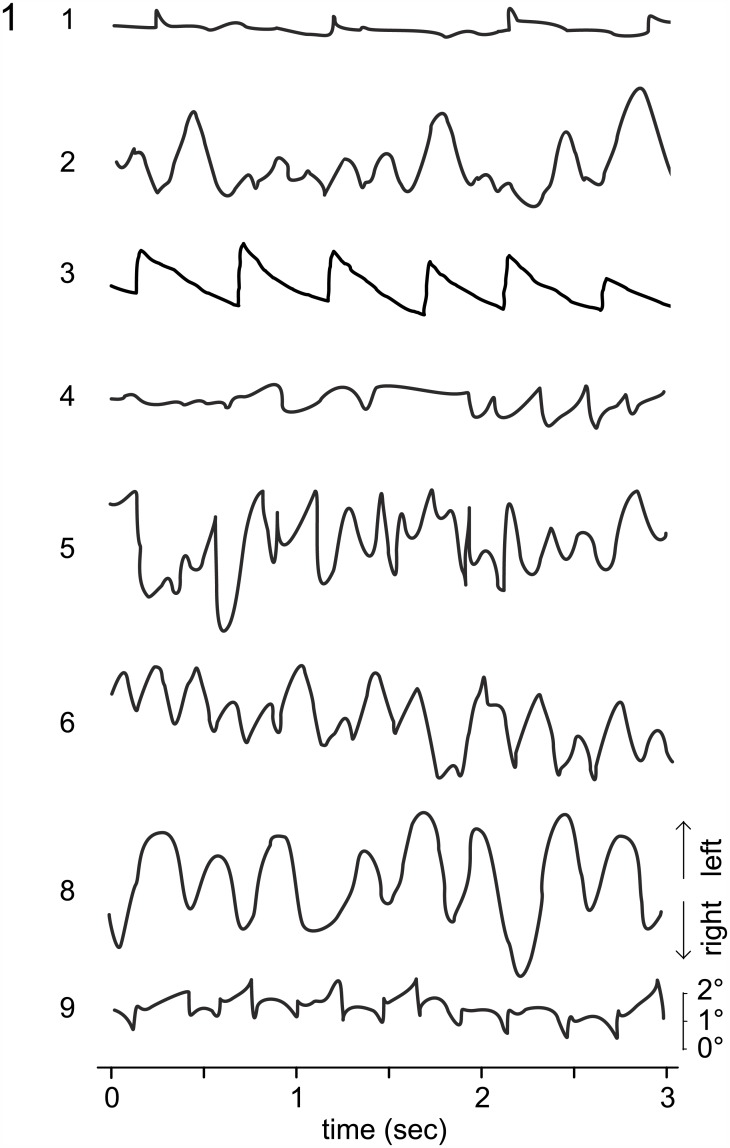
Eye movement recordings of the subjects with nystagmus. 3 seconds of horizontal eye movement recording in primary positions from subjects with nystagmus shows a variety of waveforms. Waveform of subject 3 served as template for ‘simulated nystagmus’. Missing data from subject 7. Arrows indicate direction of eye movement.

Each subject was instructed to read out loud 4 series of 40 randomly presented isolated words with 8 letters each. The words were presented with the font Helvetica and a letter size of 44 pixels (1.65°) requiring a minimal VA of 0.05 (decimal) or 1.3 logMAR. In two series, the words were presented steady in the center of the screen. In the remaining two series, the words were presented with a gaze contingent display, which moves the words in parallel to the subjects’ eye movements, thus minimizing movement due to nystagmus. The series were presented alternately with steady and gaze contingent display, with one of the two reading conditions randomly selected as the starting condition. The order of the word series was changed for every subject. As a control, healthy subjects were to read an additional fifth series with 40 moving words, thus with ‘simulated nystagmus’. For this condition the movement pattern of a 30 second recording of subject 3 was used in an infinite loop. This nystagmus displayed an amplitude of 1.97 ± 0.93 (mean ± standard deviation (SD)), a frequency of 120 per minute, no foveation period (Fig 1) and was chosen due to the most continuous appearance of waveform over time from all subjects.

Subjects were instructed to read out loud the words as rapidly and as accurately as possible as soon as they appeared on the screen. A key pressed by the subjects displayed the next word.

#### Data analysis for word reading

Word reading latency was determined by measuring the time from onset of word presentation until the first phoneme of the word was spoken (reaction time). Latencies <200 milliseconds (ms) and >2000ms were excluded as obvious outliers. For eye movement analysis, the number of fixations made from word onset until subjects pressed the key and the duration of the first fixation were assessed. For all eye movement analysis we used SR Research EyeLink Data Viewer V 1.11.1 and Microsoft Excel.

For statistical analysis a linear mixed effects model with latency, fixation count, and first fixation duration as dependent variables was used. Reading condition (‘steady’, ‘gaze contingent’, ‘simulated nystagmus’ for controls, and ‘steady’, ‘gaze contingent’ for subjects with albinism) was used as the independent variable. Subjects were used as a random effect. Latency in the steady condition was used as dependent variable to compare ‘controls’ and ‘albinism’ as independent variables. To select between different fitting models (random-intercept, random-slope, or combined) Akaike’s Information Criterion (AIC) was used and the best model by the principle ‘smaller-is-better’ was chosen. p-values are reported and p was considered as significant if p<0.05. Analyses were performed using the MIXED procedure in SPSS (IBM SPSS Statistics 21).

#### Experimental protocol for text reading

Text samples from the ‘International Reading Speed Texts’ (IReST, vision research, Germany) were used [25,26]. Each of ten texts consisted of 11 lines and 138 ± 3.6 words (mean ± SD) or 861 ± 8.8 letters (signs). All participants were instructed to read stable texts as fast as possible. Gaze contingent display for whole texts was not feasible because the gaze contingent display would also correct for the normal reading saccades and thus prevent reading. For healthy participants, the texts were presented on the computer screen in the conditions ‘steady’ and ‘simulated nystagmus’ as described above for the words. Texts were presented with a 22 pixel size (the minimal visual acuity for reading this was 1.0 logMAR) of the font Helvetica with line spacing of 1.5. In order to counterbalance for possible learning and fatigue effects and to control for variations in difficulties of the paragraphs, we pseudo-randomized the allocation of steady texts and texts read with simulated nystagmus and the order was changed for every subject. Eye movements were recorded simultaneously. Recordings were stopped manually by pressing a key as soon as readings of texts were finished.

Participants with nystagmus were to read the same texts under steady conditions from paper sheets of A3-dimensions to achieve the best visual acuity with highest reading speed. They were allowed to use their preferred reading distance as well as head position.

### Data analysis for text reading

To determine reading speed, time from text presentation until the last word was spoken (reading duration) was divided by the number of words per paragraph and the number of letters per paragraph. This resulted in number of words per second and number of letters per second.

For eye movement analysis of the healthy subjects, saccadic amplitude, number of saccades, and proportion of regressive saccades for each text was determined. To avoid inclusion of microsaccades, saccades used to change lines, and saccades unrelated to reading, we excluded all saccades <1° and >10° and all saccades in vertical direction. Additionally, fixation count, average duration of the performed fixations, and maximal fixation duration were determined.

For statistical analysis, a linear mixed effects model with reading speed (letters per second), saccadic amplitude, saccade count, number of regressive saccades, fixation count, average fixation duration, and maximal fixation duration as dependent variables was used. Reading speed in letters per second was chosen because it applies better to single word reading than words per minute and is consistent with previous reports from us [27,28] and other groups [20,29]. For controls, reading condition (‘steady’ and ‘simulated nystagmus’) was used as the independent variable. Subjects were used as a random effect. Reading speed in the steady condition was compared between controls and subjects with nystagmus. To select between different fitting models (random-intercept, random-slope, or combined) Akaike’s Information Criterion (AIC) was used and the best model was chosen by the principle ‘smaller-is-better’. p-values are reported and p was considered as significant if p<0.05. Analyses were performed using the MIXED procedure in SPSS (IBM SPSS Statistics 21).

## Results

### Subjects

The BCVA of subjects with nystagmus ranged from 0.2 to 0.9 logMAR with a mean of 0.5 logMAR. All healthy subjects had a BCVA of 0.

### Word reading

**Fig 2:**
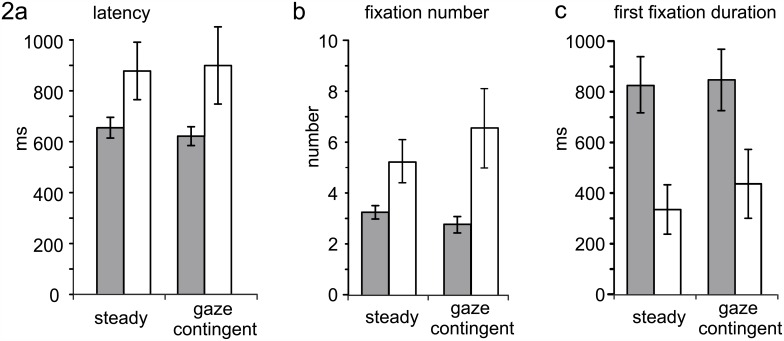
Word reading parameters for healthy controls (grey) and subjects with nystagmus (white) for the reading conditions ‘steady’ and ‘gaze contingent’. A) latency, B) fixation number, C) first fixation duration. (mean ± standard error of the mean (SEM)).

First we compared single word reading speed between subjects with nystagmus and healthy controls. We found that the word recognition time in the ‘steady’ reading condition was significantly longer in subjects with nystagmus as compared to healthy controls (albinism and nystagmus: 877.21 ± 247.43ms, controls: 684.05 ± 39.53ms; p = 0.027, Fig 2a). In subjects with nystagmus, large interindividual differences were found for all reading conditions. The analysis of eye movements showed that subjects with nystagmus made more fixations (p = 0.016) with a shorter first fixation duration (p = 0.009) than controls (Fig 2b and 2c).

**Fig 3:**
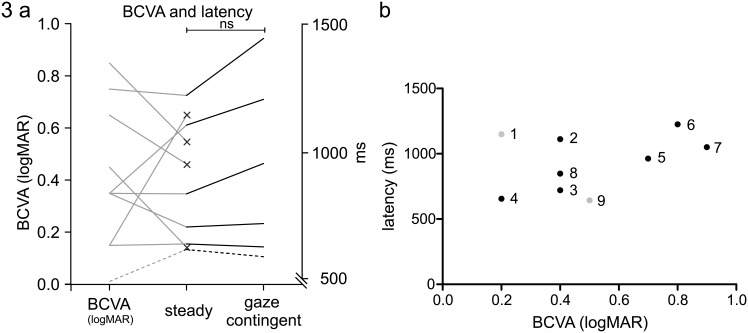
BCVA and latency for single word recognition in subjects with nystagmus. A) (x) = data of subjects only measured in steady reading conditions. Dashed line is the average of the latency from the healthy controls. B) Correlation of BCVA with latencies, grey subjects: see explanation in discussion.

Next we compared the reading parameters of participants with albinism reading ‘steady’ and ‘gaze contingent’ words. Word reading latency (p = 0.494), fixation count (p = 0.312), and first fixation duration (p = 0.743) were not different between ‘steady’ and ‘gaze contingent’ reading (Fig 3a). For detailed values see Table 2.

**Table 2:** Word reading parameters for subjects with nystagmus and healthy controls (mean ± standard deviation (SD)).

	steady	gaze contingent	simulated nystagmus	*p* value
	**albinism and nystagmus**			
**latency** (ms)	877.21±247.43	897.03±331.86	-	0.494
**fixation count**	5.11±2.51	6.55±5.79	-	0.312
**first fixation duration** (ms)	343.73±218.38	422.22±329.44	-	0.743
	**healthy controls**			
**latency** (ms)	684.05±39.53	655.22±40.69	653.76±56.62	0.498
**fixation count**	3.21±0.22	2.87±0.26	3.02±0.31	0.108
**first fixation duration** (ms)	819.36±71.04	847.98±76.60	692.17±113.19	0.047

No significant difference in latencies (p = 0.498) and number of fixations (p = 0.108) between the different reading conditions was found in healthy controls. First fixation duration was significantly different between the three conditions (p = 0.047); significance was driven by a difference between ‘gaze contingent’ and ‘simulated nystagmus’ (p = 0.034).

### Text reading

**Fig 4:**
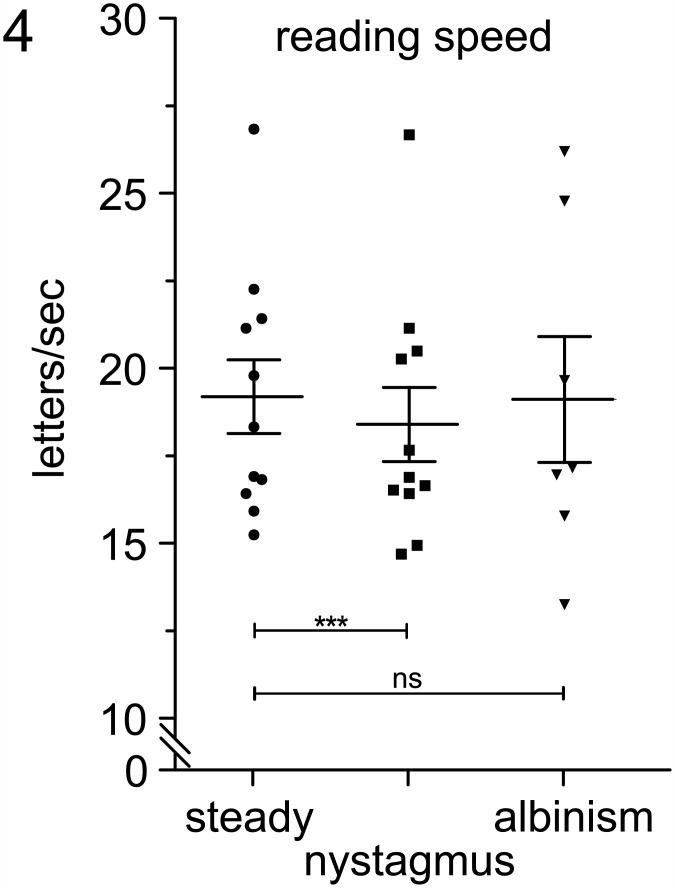
Reading speed for healthy controls in ‘steady’ reading condition, ‘simulated nystagmus’, and subjects with albinism (mean ± SEM).

In contrast to word reading, no difference of reading speed between subjects with nystagmus and controls was found in text reading (p = 0.940, Table 3). One-way ANOVA did not reveal significant differences between controls and subjects with nystagmus. The measured values lie within the reference values from the International Reading Speed Texts (IReST; 18.76±0.18 letters/s; p = 0.687) (Fig 4).

**Table 3:** Reading speed for healthy controls, albinism, and parameters for healthy controls (mean ± SD).

	steady	simulated nystagmus	albinism and nystagmus	*p* value
**reading speed** (letters/s)	19.19±3.50	-	19.66±5.22	0.940
**reading speed** (letters/s)	19.19±3.50	18.40±3.51	-	<0.001
**mean fixation duration** (ms)	185.56±25.76	216.01±27.74	-	<0.001
**max fixation duration** (ms)	704.75±157.75	794.15±161.94	-	0.023
**fixations / 10 letters**	2.42±0.45	2.28±0.51	-	0.196
**saccade amplitude** (°)	3.14±0.62	3.30±0.71	-	0.631
**saccades / 10 letters**	0.82±0.14	0.76±0.16	-	0.009
**% regressive saccades**	13.22±5.55	18.35±4.46	-	0.001

Controls showed significantly slower reading speed (fewer letters per second) in conditions with ‘simulated nystagmus’ as compared with ‘steady’ reading conditions (p<0.001).

Next, we analysed eye movements in control subjects reading steady texts and passages with simulated nystagmus. There was no difference in number of fixations (p = 0.196) between the two reading conditions, but fixation duration was significantly longer in simulated nystagmus (mean p<0.001, maximal p = 0.023). Analysis of saccades showed fewer saccades (p = 0.009) but more regressive saccades (p<0.001) in ‘simulated nystagmus’, whereas the saccadic amplitude was unchanged (p = 0.631).

## Discussion

We found that correcting nystagmus with a gaze contingent display did not improve reading speed of single words in subjects with nystagmus. This suggests that reading is not primary limited by the nystagmus. This finding is supported by the lack of different reading speeds for whole texts in affected subjects compared to controls. On the level of single word recognition, however, we found differences: Single word recognition takes longer in subjects with nystagmus than in controls. Our interpretation of this finding is that the simple perceptual task of single word recognition critically depends on sensory performance, which is impaired in subjects with nystagmus. The rate limiting step for reading an entire text, however, is more dependent on cognitive functions such as linguistic skills, understanding of content, etc.

### Word reading

Reading of single words with a gaze contingent display did not improve reading performance in subjects with nystagmus even though the latency of single word recognition was higher in subjects with nystagmus as compared to healthy subjects. This may indicate that nystagmus is not the rate limiting step even in single word recognition, and the increased latency in nystagmus as compared to controls may rather be due to the sensory deficit independent of the nystagmus, such as foveal hypoplasia and (not in this study) amblyopia. This explanation is consistent with findings from Woo et al. [20], who tested subjects with congenital nystagmus by rapid serial visual presentation (RSVP) of texts (which led to better reading performance in subjects with nystagmus) and continuous texts. They concluded that participants read at rates that are faster than the frequency of nystagmus. Westheimer et al. found that resolution threshold is not altered by movement velocities up to 2.5° in healthy individuals [30]. Thus for retinal image velocity greater than 2.5°/sec (retinal image slip), as is typically the case in nystagmus, a decrease of visual acuity and thus a sensory deficit is expected. Consistent with this prediction we found reduced single word recognition in healthy subjects with simulated nystagmus. Another explanation for this finding may be that nystagmus is insufficiently corrected by the gaze contingent display due to inaccuracies in calibration and delay from gaze detection until the display refreshes (see methods).

Interestingly, in our study, subjects 3, 4 and 9 had word-recognition latencies similar to normal (within 1 SD of the mean normal latencies), whereas subjects 2, 5, 6, and 8, all of whom have pendular nystagmus, did not. With the exception of subject 1 (whose nystagmus is very low amplitude and low frequency), it appears that the subjects with jerk nystagmus wave forms exhibited close to normal word-recognition latencies, whereas the latencies of the 4 subjects with pendular wave forms were substantially longer. Although the numbers of subjects is small, these results suggest that the nystagmus wave form might have an influence on recognition latency.

In our subjects with nystagmus, large interindividual differences were found for all reading conditions. This variability might originate from differences of the primary sensory function such as foveal hypoplasia or from different waveforms of the nystagmus, i.e. the motor function (see Fig 1). Given the close association between nystagmus and primary sensory function, our data do not allow differentation between these two possibilities. A longer foveation period allows subjects to read during this time, and thus those subjects are at an advantage over those without or with an extremely short foveation period. Subject 4 additionally shows signs of a periodic alternating nystagmus, subjects 2, 5, 6, 8 presented with predominantly pendular nystagmus, and subject 9 changed direction of nystagmus in primary position every 2 to 4 beats. We chose the nystagmus waveform from subject 3 for the ‘simulated nystagmus’ paradigm. The nystagmus of this subject has features of a manifest latent nystagmus (fusion maldevelopment nystagmus syndrome) and may therefore not be representative in the sense that this waveform has no foveation period. Given the heterogeneity of nystagmus waveforms our data do not allow the attribution of any waveform to a specific reading performance. We postulate though that using a different waveform as template would have affected the detection speed for single words. In the past, a study on healthy subjects presented with simulated infantile nystagmus showed that visual acuity was dependent not only on the duration of the foveation period, but also on the non-foveating phases [31]. Because subjects with infantile nystagmus report little or no oscillopsia in association with their nystagmus, it remains unclear whether the rapid retinal image motion during the non-foveating phases of the nystagmus waveform generates a similar degradation of visual acuity in individuals with infantile nystagmus.

Another view on impairment of single word detection in nystagmus comes from Wang and Dell’Osso, who found subjects with infantile nystagmus syndrome were ‘slow to see’ [32]. They found that patients could alter the foveation periods to discern details in the target and therefore increase the visual acuity, however, these changes resulted in a generally slower performance. As can be seen in Fig 3a & 3b, latencies correspond well to logMAR visual acuities (r = 0.7, trend to significant correlation p = 0.088, grey dots in Fig 3b not included in the correlation). The latter were two exceptions though: One subject had very long latencies despite good visual acuity. The second exception was a subject with low visual acuity but by comparison fast latencies. Exception 1, with an age of 12 years, was the youngest participant. The latency is possibly explained by a lack of reading experience. Exception 2 was the oldest participant and the most experienced reader. This most experienced subject showed very remarkable reading speeds for text reading as well (26.19 letters/s, mean 19.66 letters/s) where only one single control subject showed slightly faster reading speed with 26.83 letters/s. In our study, overall vision of the subjects with nystagmus (0.5 logMAR) was comparable with other studies (0.5 logMAR according to Kumar et al. [13] or 0.57 logMAR in a study by Mohammad et al. [33]). Another factor leading to longer latencies may be that the single words may have been presented at unfavorable times during the nystagmus, for example during a fast phase. It might therefore take the subjects with albinism part or all of an additional nystagmus cycle to recognize the word. This may explain the larger standard errors for word-recognition latency that are shown in Table 2 for the subjects with albinism compared to normal controls.

In the analysis of the reading parameters (fixation count and first fixation duration), we found significantly more fixations with shorter durations in subjects with nystagmus. This may indicate the oculomotor strategy of subjects with nystagmus. However, as we did not exclude the nystagmus fast phases, the results from the fixation analysis must be interpreted with caution. Nystagmus itself, without a reading task, is associated with more fixations.

### Text reading

In contrast to the reading latency for single words, we could not find any significant differences in text reading speed between steady reading conditions in healthy controls and subjects with nystagmus. As we used the IReST texts, we were able to compare our results with normalised reading speed values. They showed that reading speed of subjects with nystagmus and controls were within normal limits. As for single words, we found large inter-individual differences for the reading speed. The result of comparable reading speed for whole texts contrasts to the ‘slow to see’-concept from Wang and Dell’Osso [32]. Our main explanation is that the perceptual limitation from nystagmus is more relevant in single words than for an entire text, where understanding, grammar, and language become more critical and more rate limiting. Thus reading of a text requires more than reading a single word and the two things are affected differently by nystagmus. Additionally, we believe that this difference may be due to the fact that subjects with nystagmus had the possibility to hold the texts in the preferred distance and read with preferred head posture and illumination, whereas this was not allowed during single word recognition. This might explain the discrepancy with slower reading performance in single word recognition in subjects with nystagmus compared to healthy controls. The subjects with nystagmus therefore controlled when each word was imaged on the retinal region used for foveation, which may or may not have been the anatomical fovea [10]. Finally, the failure to find a significant difference in reading speed could also reflect limited statistical power because of substantial between-subject variability and the limited sample size.

‘Simulated nystagmus’ in healthy subjects however showed reduced reading speed as compared to ‘steady’ reading conditions. Our interpretation of this is that an adaptation to nystagmus is required for reaching optimal reading speed. Healthy subjects with acute new onset ‘nystagmus’ had no time to adapt. Another explanation is that individuals with infantile nystagmus syndrome (INS) or fusion maldevelopment nystagmus syndrome (FMNS) develop adaptive reading strategies with which they can control the absence or presence, timing, amplitude, and direction of nystagmus quick phases, leading to modulation of involuntary slow oscillations [32,34]. This might allow subjects with nystagmus to modulate their nystagmus while reading. This is of course not possible with our ‘simulated nystagmus’ paradigm. Another issue is that presumably many of the subjects with albinism had some degree of foveation in their waveform, while the simulated nystagmus waveform was that of a FMNS without any foveation period. Furthermore it has to be noted that all healthy subjects perceived motion/oscillopsia with simulated nystagmus. In contrast, individuals with nystagmus do not experience oscillopsia. The principal difference between these conditions is the presence of extraretinal signals for nystagmus, which do not exist when similar image motion is simulated in normal subjects.

Our findings of comparable reading speeds between healthy subjects and subjects with albinism agree with the recent findings of Barot et al. who found that maximum reading speed can be near normal in subjects with infantile nystagmus when optimal font sizes are provided, even in individuals with poor visual acuity or intense nystagmus [21]. MacDonald et al. as well found that visual impairment in albinism does not significantly impair the acquisition of normal reading skills, except for a mild correlation of decreased reading fluency [18].

Analysis of eye movement parameters during text reading in the steady condition and simulated nystagmus in healthy participants showed that in both conditions the same number of fixations were used; however, mean fixation duration was longer in simulated nystagmus. A comparable observation was made by Yang et al. who found that a text change during a fixation can increase the duration of fixation. This increased fixation duration could be the result of disrupted text processing, or from the effect of perceiving the brief visual change [35]. Analysis of the saccades showed no difference in saccadic amplitude, but interestingly, in simulated nystagmus, healthy subjects used fewer saccades, which fits well to the longer fixation duration. It is possible that they used smooth pursuit to follow the target, however, this would not explain the longer fixation duration. More likely is that the longer fixation durations and fewer saccades made by normal subjects in the simulated-nystagmus condition reflect a strategy of ‘waiting’ for words to reach the fovea, similar to the strategy of quick-phase suppression noted by Thomas et al. in some subjects with infantile nystagmus [34]. It is possible that during longer fixation when reading moving texts, larger areas with more words in the periphery could have been captured with no need for further saccades and fixations. On the other hand, in our study, the number of regressive saccades was significantly elevated (from 13.2% in steady reading conditions to 18.4% with simulated nystagmus). Whereas the value of 13.2% of regressive saccades under normal reading conditions is in line with or is even rather low compared with the amount of approximately 13–16% reported in the literature [27,36,37] or even up to 15–25% according to Rayner et al. [38,39], the measured amount during simulated nystagmus lies on the upper bound. Perhaps the moving text may have required subjects to go back and find a word they missed, thus accounting for the increase in regressions.

To summarize, we measured that moving texts in healthy controls were read with more regressive saccades and longer fixation durations, but not as might be assumed, with shorter saccades and accordingly more fixations. This is in accordance with Kanonidou et al. who found that slower reading in strabismic amblyopic patients was associated with significantly more regressive saccades and longer fixation duration, but not with changes in saccadic amplitudes [36]. Subjects with nystagmus show more fixations with shorter fixation durations during reading than normal controls, which is most likely due to the nature of the nystagmus. However, the underlying condition of nystagmus might also have affected the oculomotor control. Compared to steady reading conditions, eye movements also changed in healthy controls when reading texts in simulated nystagmus: they too showed shorter mean fixation durations and more regressive saccades in texts read with simulated nystagmus. Again we wish to point out that the analysis of saccades and of fixations must be made with caution, as nystagmus itself is associated with increased fixations and saccades independent of a reading task.

## Conclusion

In summary, our data show normal reading speed in subjects with albinism associated nystagmus, whereas the latency until a word was spoken, was significantly longer in these participants. Minimization of nystagmus with a gaze contingent display did not affect (neither improve nor reduce) the reading speed. Simulation of nystagmus in healthy subjects by showing a moving text did reduce reading speed, and the number of regressive saccades was significantly increased compared to steady reading conditions. All this argues against the nystagmus as the rate limiting factor for reading speed of text. Other sensory visual impairments associated with albinism (as for example reduced visual acuity) might be the primary causes for recognition and/or reading differences.

## Supporting Information

S1 File ‘Reading with Nystagmus, Supplementary data’ (Table A for word reading, Table B for text reading). (DOCX)
