# Supplementary material for: Nystagmus Does Not Limit Reading Ability in Albinism
Source: PLoS One. 2016 Jul 8;11(7):e0158815. doi: 10.1371/journal.pone.0158815 (PMC4938398; doi:10.1371/journal.pone.0158815)
Supplement: S1 File — (DOCX) [file pone.0158815.s001.docx]

**Reading with Nystagmus**

**Supplementary data**

Word Reading

Table A. **Word reading parameters for a) healthy controls and b) subjects with albinism and nystagmus**.

1. healthy controls

|  | **latency** (ms) | | | **fixation count** | | | **first fixation duration** (ms) | | |
| --- | --- | --- | --- | --- | --- | --- | --- | --- | --- |
| **subj**. | **steady** | **gaze contingent** | **simulated nystagmus** | **steady** | **gaze contingent** | **simulated nystagmus** | **steady** | **gaze contingent** | **simulated nystagmus** |
| 1 | 891.29 | 737.63 | 706.36 | 3.55 | 3.44 | 2.95 | 893.04 | 953.41 | 608.67 |
| 2 | 710.40 | 617.08 | 670.69 | 2.81 | 1.59 | 2.87 | 1171.28 | 1350.66 | 695.08 |
| 3 | 871.85 | 890.42 | 891.51 | 4.96 | 4.75 | 4.78 | 436.18 | 514.11 | 522.70 |
| 4 | 770.19 | 777.20 | 715.18 | 3.31 | 3.53 | 3.00 | 852.64 | 707.44 | 736.30 |
| 5 | 828.66 | 781.23 | 921.16 | 4.03 | 4.09 | 4.00 | 548.78 | 628.14 | 522.77 |
| 6 | 531.18 | 491.97 | 513.23 | 3.30 | 2.93 | 2.93 | 655.00 | 758.16 | 574.05 |
| 7 | 535.24 | 578.68 | 540.53 | 3.28 | 2.95 | 3.15 | 583.06 | 575.76 | 655.50 |
| 8 | 622.13 | 512.93 | 523.69 | 3.00 | 1.86 | 3.10 | 792.97 | 976.18 | 568.15 |
| 9 | 507.40 | 512.07 | 555.69 | 2.23 | 1.53 | 1.89 | 1194.18 | 1417.57 | 1196.20 |
| 10 | 748.76 | 711.30 | 650.03 | 3.49 | 3.41 | 3.20 | 637.54 | 516.28 | 533.08 |
| 11 | 648.09 | 627.91 | 571.34 | 3.15 | 2.42 | 2.39 | 862.25 | 778.48 | 656.74 |
| 12 | 559.65 | 595.46 | 628.57 | 1.48 | 1.60 | 2.08 | 1404.41 | 1286.84 | 1095.06 |
| mean | 684.05 | 655.22 | 653.76 | 3.20 | 2.87 | 3.02 | 819.36 | 847.98 | 692.17 |
| SD | 39.53 | 40.69 | 56.62 | 0.22 | 0.26 | 0.30 | 71.04 | 76.60 | 113.19 |

1. albinism and nystagmus

|  | **latency** (ms) | | **fixation count** | | **first fixation duration** (ms) | |
| --- | --- | --- | --- | --- | --- | --- |
| **subj**. | **steady** | **gaze contingent** | **steady** | **gaze contingent** | **steady** | **gaze contingent** |
| 2 | 1112.12 | 1211.08 | 6.54 | 10.48 | 248.44 | 92.25 |
| 3 | 720.72 | 733.92 | 2.63 | 2.31 | 705.08 | 716.03 |
| 4 | 655.97 | 644.51 | 4.14 | 2.41 | 316.83 | 744.58 |
| 6 | 1226.03 | 1444.45 | 9.23 | 16.03 | 177.83 | 140.43 |
| 8 | 848.09 | 964.36 | 5.10 | 8.21 | 188.14 | 159.69 |
| mean | 877.21 | 897.03 | 5.11 | 6.55 | 343.73 | 422.22 |
| SD | 247.43 | 331.86 | 2.51 | 5.79 | 218.38 | 329.44 |
| *Mean and SD in steady condition of all subjects:* | | | | | | |
| *1* | *1085.05* |  | *4.30* |  | *825.53* |  |
| *5* | *962.90* |  | *2.13* |  | *59.65* |  |
| *7* | *1050.22* |  | *0.99* |  | *109.65* |  |
| *9* | *643.52* |  | *4.43* |  | *132.94* |  |
| *mean* | *896.63* |  | *4.22* |  | *363.65* |  |
| *SD* | *214.35* |  | *2.50* |  | *272.05* |  |

Text Reading

Table B. **Text reading parameters for healthy controls and subjects with albinism and nystagmus**.

|  | **reading speed** (letters/s) | | **mean first**  **fixation duration** (ms) | | **max first**  **fixation duration** (ms) | | **fixations / 10 letters** | |
| --- | --- | --- | --- | --- | --- | --- | --- | --- |
| **subj**. | **steady** | **simulated nystagmus** | **steady** | **simulated nystagmus** | **steady** | **simulated nystagmus** | **steady** | **simulated nystagmus** |
| 1 | 16.83 | 16.43 | 201.3 | 234.5 | 605.8 | 920.5 | 2.34 | 2.27 |
| 2 | 15.92 | 16.52 | 243.4 | 272.6 | 832.0 | 855.0 | 2.26 | 1.98 |
| 3 | 15.24 | 14.69 | 174.4 | 178.5 | 811.6 | 771.4 | 3.11 | 3.26 |
| 4 | 21.42 | 20.50 | 187.3 | 211.5 | 616.4 | 832.4 | 2.11 | 1.97 |
| 5 | 16.92 | 16.89 | 164.2 | 193.0 | 776.2 | 600.0 | 2.89 | 2.60 |
| 6 | 16.42 | 14.95 | 145.5 | 190.4 | 590.2 | 791.4 | 3.17 | 3.02 |
| 7 | 21.15 | 20.27 | 177.4 | 218.0 | 650.0 | 804.4 | 2.25 | 1.96 |
| 8 | 22.26 | 21.14 | 188.3 | 218.8 | 573.0 | 743.2 | 2.03 | 1.92 |
| 9 | 19.79 | 17.67 | 208.6 | 251.6 | 1064.8 | 1161.8 | 2.08 | 1.98 |
| 10 | 18.33 | 16.65 | 171.1 | 201.3 | 516.8 | 660.4 | 2.62 | 2.54 |
| 12 | 26.83 | 26.67 | 179.6 | 209.7 | 715.4 | 577.0 | 1.86 | 1.62 |
| mean | 19.19 | 18.40 | 185.56 | 216.01 | 704.75 | 794.15 | 2.42 | 2.28 |
| SD | 3.50 | 3.51 | 25.76 | 27.74 | 157.75 | 161.94 | 0.45 | 0.51 |

|  | **saccade amplitude** (°) | | **saccades / 10 letters** | | **% regressive saccades** | | **reading speed** (letters/s) | |
| --- | --- | --- | --- | --- | --- | --- | --- | --- |
| **subj.** | **steady** | **simulated nystagmus** | **steady** | **simulated nystagmus** | **steady** | **simulated nystagmus** |  | **albinism** |
| 1 | 3.22 | 3.41 | 0.71 | 0.55 | 12.69 | 18.20 |  |  |
| 2 | 2.75 | 2.99 | 0.92 | 0.80 | 8.36 | 12.37 |  | 13.25 |
| 3 | 2.58 | 2.39 | 1.00 | 0.99 | 14.39 | 20.15 |  | 16.96 |
| 4 | 3.04 | 3.16 | 0.77 | 0.64 | 4.40 | 12.69 |  | 24.77 |
| 5 | 2.93 | 3.22 | 1.00 | 0.98 | 19.06 | 22.62 |  |  |
| 6 | 2.67 | 2.65 | 0.84 | 0.87 | 6.75 | 16.00 |  |  |
| 7 | 3.37 | 3.49 | 0.90 | 0.78 | 20.21 | 20.40 |  | 15.79 |
| 8 | 4.20 | 4.18 | 0.64 | 0.57 | 12.12 | 14.26 |  | 17.16 |
| 9 | 2.94 | 3.05 | 0.84 | 0.78 | 9.51 | 15.18 |  | 26.19 |
| 10 | 3.15 | 3.35 | 0.83 | 0.84 | 17.17 | 26.96 |  |  |
| 12 | 4.55 | 4.50 | 0.59 | 0.55 | 20.24 | 19.13 |  |  |
| mean | 3.22 | 3.31 | 0.82 | 0.76 | 13.22 | 18.35 |  | 19.66 |
| SD | 0.62 | 0.61 | 0.14 | 0.16 | 5.55 | 4.46 |  | 5.22 |
